# Multivariate linear time-series modeling and prediction of cerebral physiologic signals: review of statistical models and implications for human signal analytics

**DOI:** 10.3389/fnetp.2025.1551043

**Published:** 2025-04-16

**Authors:** Nuray Vakitbilir, Amanjyot Singh Sainbhi, Abrar Islam, Alwyn Gomez, Kevin Yuwa Stein, Logan Froese, Tobias Bergmann, Davis McClarty, Rahul Raj, Frederick Adam Zeiler

**Affiliations:** ^1^ Biomedical Engineering, Price Faculty of Engineering, University of Manitoba, Winnipeg, MB, Canada; ^2^ Section of Neurosurgery, Department of Surgery, Rady Faculty of Health Sciences, University of Manitoba, Winnipeg, MB, Canada; ^3^ Department of Human Anatomy and Cell Science, Rady Faculty of Health Sciences, University of Manitoba, Winnipeg, MB, Canada; ^4^ Department of Clinical Neuroscience, Karolinska Institutet, Stockholm, Sweden; ^5^ Undergraduate Engineering, Price Faculty of Engineering, University of Manitoba, Winnipeg, MB, Canada; ^6^ Undergraduate Medicine, Rady Faculty of Health Sciences, University of Manitoba, Winnipeg, MB, Canada; ^7^ Department of Neurosurgery, University of Helsinki, Helsinki, Finland; ^8^ Division of Anaesthesia, Department of Medicine, Addenbrooke’s Hospital, University of Cambridge, Cambridge, United Kingdom

**Keywords:** cerebral physiologic signals, multivariate time-series analysis, computational neuroscience, brain function modeling, statistical models, state-space models

## Abstract

Cerebral physiological signals embody complex neural, vascular, and metabolic processes that provide valuable insight into the brain’s dynamic nature. Profound comprehension and analysis of these signals are essential for unraveling cerebral intricacies, enabling precise identification of patterns and anomalies. Therefore, the advancement of computational models in cerebral physiology is pivotal for exploring the links between measurable signals and underlying physiological states. This review provides a detailed explanation of computational models, including their mathematical formulations, and discusses their relevance to the analysis of cerebral physiology dynamics. It emphasizes the importance of linear multivariate statistical models, particularly autoregressive (AR) models and the Kalman filter, in time series modeling and prediction of cerebral processes. The review focuses on the analysis and operational principles of multivariate statistical models such as AR models and the Kalman filter. These models are examined for their ability to capture intricate relationships among cerebral parameters, offering a holistic representation of brain function. The use of multivariate statistical models enables the capturing of complex relationships among cerebral physiological signals. These models provide valuable insights into the dynamic nature of the brain by representing intricate neural, vascular, and metabolic processes. The review highlights the clinical implications of using computational models to understand cerebral physiology, while also acknowledging the inherent limitations, including the need for stationary data, challenges with high dimensionality, computational complexity, and limited forecasting horizons.

## 1 Introduction

The notably high energy demand of brain cells, compared to most other bodily tissues, necessitates a constant energy supply through oxidative metabolism, and any momentary disruption in oxygen delivery can lead to severe consequences potentially resulting in brain damage or even fatality (Ainslie et al., 2007). Sustaining oxygen availability relies on an intricate and resilient hemodynamic regulation system called cerebral autoregulation, which modulates cerebral blood flow (CBF) in response to variations in systemic supply, such as blood pressure and oxygen saturation, and cerebral demand, particularly energy consumption linked to neuronal activity (Liu et al., 2019; Kostoglou et al., 2014). Dysfunction in cerebral regulatory mechanisms is common in various disease states, making the monitoring of cerebral oxygenation and metabolism an essential aspect of neurocritical care management (Chen et al., 2006). The dynamics of cerebral autoregulation, however, vary significantly across different disease conditions, often impairing the brain’s ability to maintain stable blood flow and oxygenation in response to changes in systemic pressure. This monitoring, in turn, allows for the acquisition of a wide range of cerebral physiologic signals in high temporal resolutions allowing for the implementation of sophisticated analytical techniques (Katsogridakis et al., 2016).

Cerebral physiologic signals encapsulate intricate neural, vascular, and metabolic activities within the brain, offering insight into the dynamic and multifaceted nature of cerebral function (Kuo et al., 1998). Continuous cerebral physiologic signals, such as intracranial pressure (ICP), cerebral autoregulation, and brain tissue oxygenation (PbtO_2_), are readily available from patients with neural injuries and those critically injured in intensive care units. Thoroughly understanding and analyzing these signals is crucial for comprehending the complexities of cerebral processes, which enables the identification of intricate patterns and the accurate pinpointing of anomalies (Peng et al., 2008; Zeiler et al., 2017). Thus, the development of computational models of cerebral physiology plays a crucial role in exploring the connections between measurable signals and the underlying physiological state.

In this narrative review, we explore the landscape of time series modeling and prediction of continuous cerebral physiology, focusing on the nuanced power of multivariate statistical models. Time series analysis serves as a fundamental tool in uncovering hidden patterns within sequential data. In time series modeling and prediction, multivariate models are used as versatile tools capable of capturing the dynamic relationships and interactions across various cerebral parameters simultaneously (Martins et al., 2020). Unlike univariate models, which may oversimplify the intricacies of cerebral physiology, multivariate models consider the interdependence of signals, providing a holistic representation of the brain’s dynamic state (Peng et al., 2008; Chacon et al., 2011).

Multivariate vector-based autoregressive (AR) models, such as vector autoregressive (VAR) models in the context of cerebral physiology, play a pivotal role in capturing the intricate dynamics of interrelated variables. These models operate by considering multiple time series simultaneously, with each variable representing a specific aspect of cerebral function (Scherrer et al., 2019). Through the estimation of lagged relationships among these variables, VAR models reveal how changes in one component influence others within the system over time. The core principle of vector-based models lies in their ability to represent the dynamic interplay and feedback loops inherent in complex physiological systems (Olson et al., 2020). By incorporating the temporal dependencies among multiple variables, these models provide a more nuanced understanding of the interactions between the cerebral processes (the time-based relationships). The estimation process involves determining coefficients that characterize the strength and direction of the relationships between variables, allowing for the prediction of future states based on past observations (Zivot and Wang, 2006). Additionally, multivariate AR models offer integration into deep learning-based methods, enhancing their capabilities for data prediction and statistical analysis (He et al., 2023).

**FIGURE 1:**
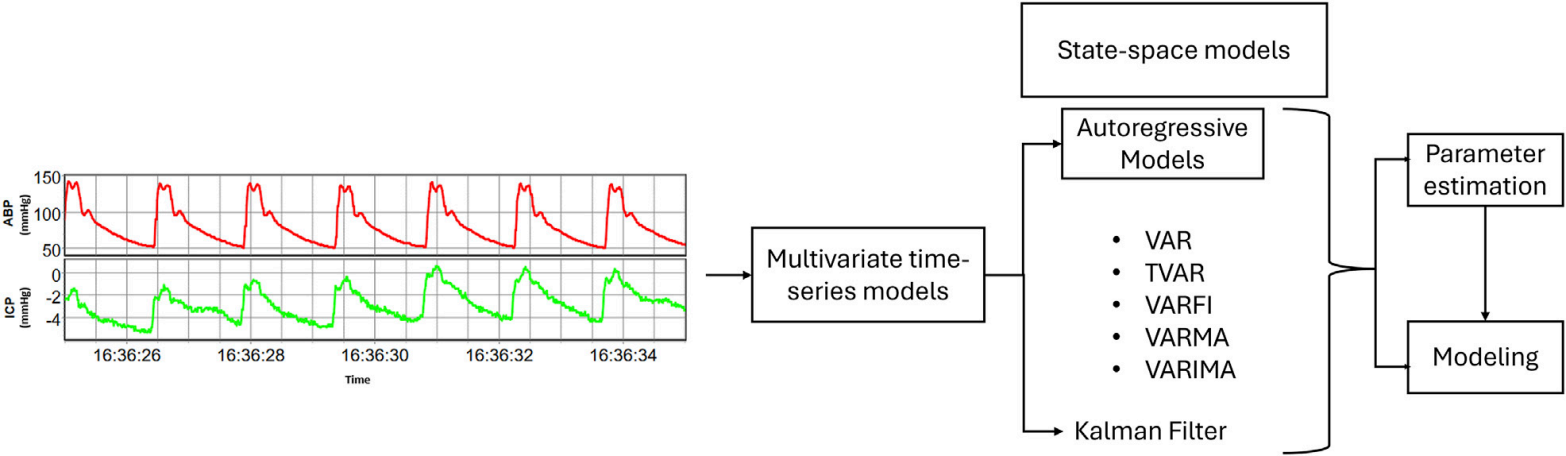
A schematic overview illustrating the pathway from raw data to final modeling.

Other multivariate state-space models, such as Kalman filter, are designed to represent and capture the evolving dynamics of a system over time (Ferreira et al., 2022). Unlike multivariate AR models that focus on relationships among observed variables, state-space models introduce the concept of unobservable states, representing latent processes that influence the observed signals (Aoki, 1990). These models consider that there are underlying hidden factors driving the observed data. The fundamental idea behind state-based models is to estimate these hidden states by combining information from the observed signals and the dynamic evolution of the system (Hamilton, 1994). They operate through a two-fold process: the state equation, describing how the system evolves over time, and the observation equation, detailing how the unobservable states contribute to the observed signals. By iteratively updating the estimates of both states and parameters, state-space models offer a comprehensive framework for modeling the intricate temporal dependencies within cerebral physiologic signals (Aoki, 1990; Hinrichsen and Holmes, 2009). Figure 1 provides a concise overview of the pathway from collected raw data to modeling using multivariate time-series models, illustrating the main steps involved in the process.

This review seeks to offer a comprehensive exploration of widely employed multivariate statistical models, namely, multivariate AR models and the Kalman filter, while intentionally excluding machine learning approaches such as Gaussian processes to focus on traditional statistical methodologies. Through a detailed examination of their operational principles and mathematical formulations, this narrative review aims to elucidate the intricacies inherent in these modeling approaches. Additionally, the discussion will extend beyond theoretical foundations to delve into the practical applications and clinical significance of these models. This review aims to provide a nuanced understanding that bridges the gap between theoretical concepts of the multivariate statistical models and their implications with respect to cerebral physiology.

## 2 Multivariate state-space models

State-space models comprise state variables, observation variables, and a set of equations governing their dynamic interactions (Hamilton, 1994). State-space modeling emphasizes the existence of unobserved or hidden states that influence the observed variables (Holmes et al., 2012). The mathematical formulation involves transition equations that describe the evolution of the system’s state over time, coupled with observation equations establishing the relationship between the state and observed variables (Aoki, 1990). State-space models come in various forms, including linear, nonlinear, discrete-time, and continuous-time models, each tailored to specific applications. The following sub-sections focus on the linear state-space models that are employed in cerebral physiology analysis, which is also summarized in Table 1.

**TABLE 1:** The summary of the linear state-space models.

Model	Definition	Advantages	Disadvantages and limitations
VAR	Describes the linear relationship between multiple variables by regressing each variable on its own lagged values and the lagged values of all other variables in the system	- Capturing dynamic interaction - Modeling temporal relationships	- Requirement for stationary data - Sensitivity to model specification - Challenges in determining the appropriate lag length - Potential overfitting with high-dimensional data
TVAR	Allows the parameters to vary over time, thereby capturing the time-varying nature of the relationships among variables	- Capturing time-varying dynamics - Improving forecasting accuracy - Detecting transient events - Exploring dynamic interactions	- Difficulties in interpreting time-varying coefficients due to model complexity - Computational intensity - Challenges in model selection - Adequate data requirements - Risk of overfitting due to model flexibility
VARFI	Combines VAR models with FI techniques to describe time series data that exhibit long memory or long-range dependence allowing for non-integer differencing orders	- Capturing dynamic responses - Incorporating external factors and interventions - Assessing the overall impact of long-term correlations - Ensuring dependable performance with short time series - Identifying intervention effects	- Requirement for stationary data - Sensitivity to model specification - Challenges in determining appropriate lag length and fractionally differencing parameters - Potential overfitting with high-dimensional data - Difficulty in interpreting causal relationships among variables - Limited forecasting horizon
VARFIMA	Models multivariate time-series with both short and long-range dependencies, making it ideal for capturing memory effects in complex systems	- Capturing long range dependencies - Handling fractional differencing - Improving predictive accuracy for time-series with long-memory characteristics	- High computational cost due to estimating of fractional differencing parameters - Parameter estimation challenges - Requires long time-series data to capture long-range dependencies - Interpretability issues due to fractional integration
VARMA	Combines VAR and MA to capture the temporal dependencies and stochastic elements inherent in time series data	- Capturing dynamic interactions and temporal dependencies - Incorporating lagged effects - Assessing intervention effects	- Requirement for stationary data - Substantial computational resources and complexity especially with increasing variables and lags
VARIMA	Incorporates ordered differencing to AR and MA to achieve stationarity in the multivariate time series data, which is particularly useful when dealing with non-stationary time series	- Capturing temporal dynamics - Accounting for non-stationarity - Providing forecasting capabilities - Assessing intervention effects	- High computational cost - Identification challenges - Stationarity assumption - Interpretability complexity - Risk of overfitting - Limited forecasting horizon - Model specification requirements
Kalman filter	Estimates and optimizes linear dynamic systems recursively in the presence of noise	- Estimating unobserved physiological states - Modeling dynamic processes - Reducing noise in measurements - Integrating data from multiple sources, enable real-time monitoring, and predict future physiological states	- Model assumptions (linearity, Gaussian noise, known model parameters) - Sensitivity to model mismatch - Computational complexity - Initialization challenges - Lack of robustness to outliers - Limited forecasting capability

FI, fractional integration; MA, mean average; TVAR, time-varying vector autoregressive; VAR, vector autoregressive; VARFI, vector autoregressive fractionally integrated; VARFIMA, vector autoregressive fractionally integrated moving average; VARIMA, vector autoregressive integrated moving average; VARMA, vector autoregressive moving average.

### 2.1 Vector autoregressive (VAR) models

AR models represent a fundamental class of time series models that play a pivotal role in understanding and predicting sequential data patterns. In essence, these models capture the idea that each observation in a time series is linearly dependent on its own past values. This singular focus on the relationship between a variable and its own lagged values provides a powerful framework for modeling temporal dependencies and capturing the inherent autocorrelation present in time series data (Olson et al., 2020). However, AR models have inherent limitations in capturing interdependencies among multiple variables. Hence, extending beyond the analysis of a single variable involves the utilization of multivariate modeling through models such as VAR, and vector autoregressive moving average (VARMA) models. These models offer a nuanced perspective that enables the exploration of how changes in one component influence others within a system (Scherrer et al., 2019; Zivot and Wang, 2006).

VAR models represent a natural extension of AR models to accommodate multiple parallel time series. The VAR model is particularly valuable when analyzing systems where several variables interact and influence each other over time. In essence, a VAR model consists of a system of dynamic equations, wherein each variable is regressed on its own lagged values and the lagged values of all other variables in the system allowing for the simultaneous consideration of interdependencies among multiple variables and capturing the intricate dynamics within a system (Zivot and Wang, 2006). The formulation for a VAR model of order 'p' (VAR(p)) encapsulates the relationships and dependencies among the variables, providing a versatile tool for projecting time-series variables and understanding the dynamic evolution of multivariate time series data. The order *p* determines the number of lagged observations included in the model. Mathematically, VAR(p) can be formulated as given in Equation 1, where Y_t_ is an n-dimensional vector of endogenous variables at time t, A terms are $\left(n\times n\right)$ coefficient matrices capturing the lagged effects, ε_t_ is a vector of white noise disturbances, and c is an n-dimensional constant term (Toda and Phillips, 1994).

$$Y_{t}=c+\sum_{i=1}^{p}A_{i}Y_{t-i}+\varepsilon_{t}$$ (1)

Each *A*_i_ matrix captures the contemporaneous relationships among the variables. The estimation of a VAR model involves determining the coefficients in these matrices which allows for the analysis of the dynamic interactions among variables using techniques such as least squares or maximum likelihood estimation (Zivot and Wang, 2006). However, when few data samples are available, VAR models can also be identified using penalized regression techniques, which help address overfitting and improve model stability (Antonacci et al., 2024a). VAR models assume linearity, stationarity, and often normality of residuals, and their effectiveness may vary depending on the characteristics of the data being analyzed (Toda and Phillips, 1994).

VAR models prove invaluable for its ability to capture dynamic interactions, model temporal relationships, and assess causality within multivariate time-series data. As an essential component of multivariate analysis, VAR models contribute to uncovering network interactions, identifying functional connectivity patterns, and enhancing sensitivity to subtle changes in brain activity.

### 2.2 Time-varying autoregressive (TVAR) model

Time-varying autoregressive (TVAR) model extends the traditional VAR model by allowing the parameters to vary over time, thereby capturing the time-varying nature of the relationships among variables (Haslbeck et al., 2021; Oikonomou et al., 2007). A typical TVAR model can be expressed as shown in Equation 2, where *Y*_*t*_ is a *p*-dimensional vector time-series at time *t*, *β* *_i,t_* are the coefficient matrices corresponding to each lag i varying over time, *ε_t_* is a *p*-dimensional vector of error terms assumed to be normally distributed with mean zero and covariance matrix (Σ*_t_*), allowing for time-varying volatility (Haslbeck et al., 2021).

$$Y_{t}=\sum_{i=1}^{p}\beta_{i,t}Y_{t-i}+\varepsilon_{t}$$ (2)

Estimating TVAR models involves estimating Σ*_t_* and *β_i_* which captures the dynamic relationships among variables over time (Guo et al., 2022). Each element of *β_i_* represents the coefficient of the corresponding lagged variable at time *t*. These coefficients are allowed to change over time, reflecting fluctuations in the relationships among variables (Guo et al., 2022). There are various methods for estimating TVAR models, including Kalman filtering, rolling window estimation, and Bayesian techniques (Haslbeck et al., 2021; Oikonomou et al., 2007; Omidvarnia et al., 2011). Additionally, least mean square methods and their recursive counterparts provide alternative approaches for estimating TVAR models, particularly in scenarios requiring adaptive filtering or online learning (Antonacci et al., 2024a). TVAR models allow capturing time-varying dynamics, improving forecasting accuracy, detecting transient events, and exploring dynamic interactions, offering valuable insights into the dynamic nature of cerebral function (Haslbeck et al., 2021; Oikonomou et al., 2007).

### 2.3 Vector autoregressive fractionally integrated (VARFI) model

Vector autoregressive fractionally integrated (VARFI) framework is a time series modeling technique that combines VAR models with fractional integration (FI) techniques. FI is used to describe time series data that exhibit long memory or long-range dependence allowing for non-integer differencing orders, which enables capturing long memory properties in the data, unlike traditional integer-order differencing (Pinto et al., 2021). The VARFI framework combines these two concepts by incorporating fractional integration into the VAR model allowing the model to capture both the linear interdependencies among multiple time series variables and the long memory properties exhibited by the data (Martins et al., 2020; Balboa et al., 2021). The VARFI process is depicted in Equation 3 where *L* refers to back-shift operator (*L^i^X_n_ = X_n-i_*), *A(L)* represents VAR polynomial of order *p*, *X_n_* is the zero-mean stationary multivariate stochastic process, and ε_t_ represents the uncorrelated Gaussian innovations (Martins et al., 2020; Pinto et al., 2021).

$$A\left(L\right)diag\left(\nabla^{d}\right)X_{t}=\varepsilon_{t}$$ (3)

${\left(1-L\right)}^{d_{i}}$ in Equation 4 refers to fractional differencing operator with i = 1, 2, 3.

$$diag\left(\nabla^{d}\right)=diag\left[{\left(1-L\right)}^{d_{i}}\right]$$ (4)

VAR model with polynomial order p is then represented by Equation 5, where *I_M_* refers to the identity matrix of size *M* where *M* represents the number of endogenous variables in the system.

$$A\left(L\right)=I_{M}-\sum_{i=1}^{p}A_{i}L^{i}$$ (5)

The parameter *d = (d_R_*, *d_S_*, *d_H_*) dictates the long-term characteristics of the process *X_i_*, while the coefficients of *A(L)* describe its short-term dynamics. By approximating a VARFI(p, d) model with a finite-order VAR(p + q) process, VARFI models prove advantageous for analyzing time series data that commonly exhibit both multivariate dependencies and long memory properties (Pinto et al., 2021).

VARFI models are capable of incorporating external factors and interventions, capturing dynamic responses, assessing the overall impact of long-term correlations, ensuring dependable performance with short time series, and identifying intervention effects (Balboa et al., 2021; Pinto et al., 2022a), thus providing valuable insights into cerebral function under varying conditions.

### 2.4 Vector autoregressive fractionally integrated moving average (VARFIMA) model

The Vector Fractionally Integrated Autoregressive Moving Average (VARFIMA) model extends the traditional VARMA model by incorporating fractional differencing, allowing for long memory behavior in multivariate time-series data (Ehouman, 2020). This is particularly useful for analyzing processes that exhibit long-range dependencies and slow decay in autocorrelations, such as physiological signals and economic time series. The general form of VARFIMA (p,d,q) model is given in Equation 6, where Y_t_ is a n-dimensional vector of time-series observation at time t, Φ(*L*) is a $\left(n\times n\right)$ matrix polynomial in the lag operator *L*, (1-*L*)*^d^* is the fractional differencing operator with a diagonal differencing matrix, θ(L) is a $\left(n\times n\right)$ matrix polynomial in *L*, and *ε_t_* is a white noise vector with mean zero and covariance matrix ∑.

$$\Phi \left(L\right){\left(1-L\right)}^{d}Y_{t}=\theta \left(L\right)\varepsilon_{t}$$ (6)

The fractional differencing operator (1-*L*)*d* is defined using the binominal expansion given in Equation 7, where Γ (∙) is the gamma function which allows for non-integer values of *d*, capturing long memory behavior in time-series.

$${\left(1-L\right)}^{d}=\sum_{j=0}^{\infty }\frac{\Gamma \left(j-d\right)}{\Gamma \left(-d\right)\Gamma \left(j+1\right)}L^{j}$$ (7)

By incorporating fractional differencing, VARFIMA provides a more flexible framework for modeling multivariate time series with long-memory characteristics compared to standard VARMA models.

### 2.5 Vector autoregressive moving average (VARMA) model

VARMA models represent a sophisticated extension of time series analysis that combines the strengths of both AR and moving average (MA) processes. VARMA models provide a powerful framework for capturing the temporal dependencies and stochastic elements inherent in time series data (Scherrer et al., 2019). Mathematically, VARMA(p, q) model can be expressed as given in Equation 8, where *Y_t_* is a vector of endogenous variables at time *t*, *A* terms are coefficient matrices capturing the lagged effects, *ε_t_* is a vector of white noise disturbances, c is a constant term, *B* terms are coefficient matrices capturing the moving average effects, and *ε_t-i_* represent the lagged white noise disturbances.

$$Y_{t}=c+\sum_{i=1}^{p}A_{i}Y_{t-i}+\varepsilon_{t}+\sum_{i=1}^{q}B_{i}\varepsilon_{t-i\;}$$ (8)

The *p* parameter represents the order of the AR component, while *q* represents the order of the MA component. The selection of the appropriate order (p, q) is crucial for the model’s accuracy and is often determined through model selection techniques. The parameters of the VARMA model are estimated using methods such as maximum likelihood estimation. VARMA models are particularly useful for capturing the interdependencies and dynamic interactions among multiple time series variables.

VARMA models extend the capabilities of VAR models by incorporating AR and MA components for exploration of temporal dependencies and stochastic processes within cerebral physiologic data. VARMA models are, similar to VAR models, capable of capturing dynamic interactions among multiple brain regions, but they can additionally incorporate the impact of past disturbances on the current state of the system (Nadalizadeh et al., 2023). This integration allows for a more comprehensive examination of the temporal dynamics of brain signals, considering both the inherent autocorrelation and the influence of random disturbances.

### 2.6 Vector autoregressive integrated moving average (VARIMA) model

VARIMA models are an extension of the univariate autoregressive integrated moving average (ARIMA) models to handle multiple time series variables simultaneously (Rusyana et al., 2020). In a VARIMA model, each variable in the system is treated as a linear function of its own past values, the past values of all other variables in the system, and possibly the past values of some white noise error terms. VARIMA models incorporate differencing to achieve stationarity in the time series data, which is particularly useful when dealing with non-stationary time series (Rusyana et al., 2020; Anderson, 1977). The general form of a VARIMA(p, d, q) model is expressed as given in Equation 9, where *Y*_*t*_ is a vector of endogenous variables at time *t*, εt is a vector of white noise disturbances, *c* is a constant term, *L* is a lag operator, Φ*_i_* are the autoregressive parameters, Θ*_i_* are the moving average parameters, *d* is the order of differencing (Olson et al., 2020). The notations *p*, *d* and *q*, similar to that in an ARIMA model, refer to the order of the AR component, the order of differencing needed to make the series stationary, and the order of MA component, respectively (Anderson, 1977).

$$\left(1-\sum_{i=1}^{p}\Phi_{i}L^{i}\right){\left(1-L\right)}^{d}Y_{t}=c+\left(1+\sum_{i=1}^{q}\Theta_{i}L^{i}\right)\varepsilon_{t}$$ (9)

VARIMA models are particularly useful for time series data with trends, as the integrated component helps in detrending the series (Rusyana et al., 2020). Similar to other vector AR models, the estimation and forecasting procedures for VARIMA models involve techniques like maximum likelihood estimation and can be more complex than those for univariate ARIMA models.

VARIMA models consider both AR and MA effects, as well as trends in the data providing a comprehensive approach to modeling and understanding the temporal dynamics of cerebral physiologic data. Through techniques like granger causality and impulse response function analyses, multivariate AR models enable the investigation of directional influences, shedding light on the causal relationships between different brain areas (Manomaisaowapak et al., 2022; Barnett and Seth, 2015).

### 2.7 Kalman filter

The Kalman filter is an algorithm used for recursive estimation and optimization of linear dynamic systems in the presence of noise (Maybeck et al., 1990). The filter operates by combining predictions from a mathematical model of the system with real-world measurements to produce accurate and reliable estimates of the system’s state (Welch, 1997; Barton et al., 2009). The Kalman filter tries to estimate the state *x* in a discrete-time controlled process using the linear stochastic difference equation given in Equation 10 where *x_t_* is the state at time-step *t*, *A* is the state transition matrix, B is the control input matrix, ut is the control input, and wt is the process noise (Welch, 1997).

$$x_{t}=Ax_{t-1}+Bu_{t}+w_{t}$$ (10)

The measurement equation that is used to relate the observed measurements to the underlying state of the system is represented as given in Equation 11, where zt is the measurement at time t, H is the measurement matrix, and vt is the measurement noise. The measurement equation defines how the true state influences the measurements that are obtained from the real-world system (Kim et al., 2019). The measurement equation plays a crucial role in the update step of the Kalman filter, where it helps refine the estimate of the system’s state based on the comparison between the predicted measurements and the actual measurements.

$$z_{t}=Hx_{t}+v_{t}$$ (11)

Two stages make up the Kalman filter algorithm, namely, prediction and update. In the prediction step, the Kalman filter predicts the current state, Equation 12 where ${\hat{x}}_{t}$ refers to the predicted state, and covariance, Equation 13 where *P_t_* is the predicted covariance, and the *Q* is the process noise covariance, based on the previous state estimate and covariance, the state transition matrix, and the process noise (Kim et al., 2019).

$${\hat{x}}_{t}=A{\hat{x}}_{t-1}+Bu_{t}$$ (12)

$$P_{t}=AP_{t-1}A^{T}+Q$$ (13)

In the update step, the Kalman gain (denoted as *K_t_*), estimated using Equation 14 where *R* represents the measurement noise covariance, is applied to update the state as per Equation 15. The covariance is also updated using Equation 16, where *P_t_* signifies the updated covariance, and *I* is the identity matrix. This update is performed based on a comparison between the predicted values and the actual measurement.

$$K_{t}=P_{t-1}H^{T}{\left(HP_{t-1}H^{T}+R\right)}^{-1}$$ (14)

$${\hat{x}}_{t}={\hat{x}}_{t-1}+K_{t}\left(z_{t}-H{\hat{x}}_{t-1}\right)$$ (15)

$$P_{t}=\left(I-K_{t}H\right)P_{t-1}$$ (16)

The Kalman filter continuously iterates through the prediction and update steps as new measurements become available, providing an optimal estimate of the system’s state even in the presence of noise (Welch, 1997).

The Kalman filter can accurately estimate unobserved physiological states, model dynamic processes, reduce noise in measurements, integrate data from multiple sources, enable real-time monitoring, and predict future physiological states (Nadalizadeh et al., 2023; Rajabioun et al., 2017; Sun et al., 2008; Azzalini et al., 2023), proving its importance in cerebral physiologic signal analysis.

## 3 Clinical relevance

In the realm of cerebral signal analysis, multivariate time-series analysis is crucial for simultaneously examining the spatial and temporal dynamics of cerebral physiological signals to study how different brain regions interact over time, consequently, capturing the complexity of neural processes that cannot be fully understood with univariate approaches (Kostoglou et al., 2014). Multivariate analysis allows assessment of the correlations and functional connectivity patterns between signals, helping to uncover network interactions and the coordination of brain activity (Salvador et al., 2020). Multivariate analysis is also more sensitive to subtle changes in brain function which is crucial for detecting early signs of neurological disorders, monitoring treatment effects, or understanding the impact of interventions on brain function (Brier et al., 2022; Gessell et al., 2021).

Vector-based models, with their multivariate nature, have the ability to capture dynamic interactions, model temporal relationships, assess causality, and provide valuable insights into the complex dynamics of brain activity over time (Ferreira et al., 2022; Aoki, 1990; Triantafyllopoulos and Triantafyllopoulos, 2021). Multivariate time series models play a crucial role in life sciences, providing powerful tools to analyze complex dynamics across animal and human populations, offering enhanced classification performance compared to simpler methods, such as univariate time series models, enabling researchers to discern subtle patterns in physiological signals such as EEG signals (Oikonomou et al., 2007; Omidvarnia et al., 2011; Nadalizadeh et al., 2023; Anderson, 1977; Rajabioun et al., 2017; Samdin et al., 2013; Pascucci et al., 2020; Hart et al., 2021; Jajcay and Hlinka, 2023; Kamiński et al., 1997; Lie and van Mierlo, 2017). They excel in capturing shared dynamics among individuals and populations, shedding light on similarities in physiological processes within and across groups.

Moreover, multivariate models facilitate the automatic assessment of critical physiological parameters, allowing for a deeper understanding of regulatory mechanisms such as cerebral autoregulation (Pinto et al., 2022b; Schäck et al., 2018; Jachan et al., 2009). Additionally, multivariate AR models serve as valuable tools for mitigating data overload by reducing data resolution, aiding in the integration of high-resolution cerebral data into predictive models, such as neural networks (Thelin et al., 2020), and enhancing utility in clinical decision-making processes. By uncovering intricate neural dynamics underlying cognitive processes and serving as tools for data resolution reduction, these models provide valuable insights into brain function, functional connectivity patterns, and the integration of high-resolution cerebral signal monitoring data into trajectory models.

Furthermore, multivariate modeling techniques enhance the analysis of EEG recordings by improving signal quality and reducing noise, leading to more accurate interpretations of brain activity and functions (Oikonomou et al., 2007). They also enable the detection of rapid changes in connectivity patterns, providing valuable information about brain network dynamics and the functions that emerge from these networks (Omidvarnia et al., 2011). Additionally, these models offer insights into cerebrovascular dynamics and the relationship between physiological variables such as ICP, mean arterial pressure, and brain oxygenation, contributing to our understanding of conditions like traumatic brain injury and their effects on brain function (Thelin et al., 2020; Zeiler et al., 2020; Zeiler et al., 2021; Valdés-Sosa et al., 2005; Antonacci et al., 2020; Antonacci et al., 2021a).

Moreover, penalized regression techniques for identifying VAR models have shown particular promise in brain-computer interface applications, where such methods are used to enhance model robustness and performance with limited data samples (Antonacci et al., 2024a). These applications should be highlighted in the context of clinical relevance, emphasizing their potential to translate complex cerebral signal analysis into actionable insights for neurological and clinical applications.

Additionally, it is worth noting that VARFIMA, currently, has not been widely applied in cerebral physiology modelling research, despite its potential advantages. Given its ability to capture both short and long-range dependencies in multivariate time-series data, VARFIMA could offer a more nuanced representation of cerebral physiologic signals, particularly in scenarios where fractional differencing can better model effects in autoregulatory indices such as pressure reactivity index (PRx). Integrating VARFIMA into cerebral physiology studies could enhance trend analysis and predictive modeling, offering a valuable framework for understanding complex neural interactions over varying temporal resolutions.

In summary, multivariate time series models are indispensable tools for studying complex physiological phenomena, offering valuable insights into brain function, cerebral dynamics, and neurological disorders across diverse populations. Table 2 presents the studies utilizing linear multivariate state-space models for analyzing various cerebral physiological signals. Majority of the studies focused on EEG signal analysis (Oikonomou et al., 2007; Omidvarnia et al., 2011; Nadalizadeh et al., 2023; Anderson, 1977; Rajabioun et al., 2017; Samdin et al., 2013; Pascucci et al., 2020; Hart et al., 2021; Jajcay and Hlinka, 2023; Kamiński et al., 1997; Lie and van Mierlo, 2017; Antonacci et al., 2023; Pagnotta and Plomp, 2018; Astolfi et al., 2008; Antonacci et al., 2024b; Antonacci et al., 2021b; Endemann et al., 2022; Milde et al., 2010), with a few focused on other cerebral physiology such as ICP (Pinto et al., 2022b; Schäck et al., 2018; Jachan et al., 2009; Thelin et al., 2020; Zeiler et al., 2020; Zeiler et al., 2021; Swiercz et al., 1998; Swiercz et al., 2000; Gomez et al., 2023a; Gomez et al., 2023b) for tasks ranging from assessment of physiological dynamics (Oikonomou et al., 2007; Jajcay and Hlinka, 2023; Kamiński et al., 1997), connectivity analysis (Omidvarnia et al., 2011; Rajabioun et al., 2017; Pascucci et al., 2020; Lie and van Mierlo, 2017), transfer entropy estimation (Pinto et al., 2022b), classification or prediction and pattern recognition (Nadalizadeh et al., 2023; Samdin et al., 2013; Zeiler et al., 2020; Zeiler et al., 2021; Swiercz et al., 1998; Swiercz et al., 2000; Anderson et al., 1998). Majority of these studies have utilized VAR model (Nadalizadeh et al., 2023; Anderson, 1977; Hart et al., 2021; Jajcay and Hlinka, 2023; Kamiński et al., 1997; Lie and van Mierlo, 2017; Jachan et al., 2009; Pagnotta and Plomp, 2018; Antonacci et al., 2024b; Antonacci et al., 2021b; Gomez et al., 2023a; Gomez et al., 2023b). Kalman filter was the second most utilized multivariate model (Oikonomou et al., 2007; Omidvarnia et al., 2011; Nadalizadeh et al., 2023; Rajabioun et al., 2017; Pascucci et al., 2020; Lie and van Mierlo, 2017; Schäck et al., 2018; Pagnotta and Plomp, 2018).

**TABLE 2:** The studies employing linear multivariate state-space models for various cerebral physiologic signal analysis.

Study	Study group	Multivariate model	Studied cerebral physiology	Significance of the model in the study
Astolfi et al. (2008)	Healthy participants	TVAR	Test-state EEG signals	The proposed model was able to correctly estimate the changes in connectivity relationships between cortical areas of human brain
Anderson et al. (1998)	Healthy participants	VAR	Test-state EEG signals	Better classification performance was observed with the coefficients extracted with VAR model compared to univariate AR model
Antonacci et al. (2021b)	Healthy participants	VAR	Test-state EEG signals	The results showed the importance of β EEG waves in analyzing multivariate interactions during motor execution and imagery tasks
Antonacci et al. (2023)	Healthy participants	TVAR	Resting-state EEG signals	Better tracking of transient pathways and spatio-temporal changes in physiological systems was observed with the TVAR model compared to stationary models
Antonacci et al. (2024b)	Healthy participants	VAR	Task-state EEG signals	The results showed that integrating advanced information theory with EEG source reconstruction enables anatomically localized analysis of complex functional interactions
Endemann et al. (2022)	Neurosurgical patients	VAR	Resting-state EEG signals	Long data segments enabled the successful estimation of high-dimensional VAR models, yielding plausible connectivity profiles
Gomez et al. (2023b)	Moderate-to-severe TBI patients	VAR	ICP, rSO2 Other; ABP	The changes in ICP and rSO2 responding to an impulse change in ABP was examined using a VAR model, aiming to identify similarities
Gomez et al. (2023a)	Moderate-to-severe TBI patients	VAR	ICP, PbtO2, rSO2 Other; ABP	Impulse-response function plots drawn based on VAR model illustrated the changes in PbtO2 and rSO2
Hart et al. (2021)	Healthy twin pairs	VAR with Markov switching	Resting-state EEG signals	The suggested model demonstrated the capacity to acknowledge that various epochs originating from a single participant would exhibit identical microstate dynamics, while also noting the existence of shared microstates among twins
Jachan et al. (2009)	Uni- or bilateral internal carotid artery stenosis or occlusion patients	VAR	CBFv Other; ABP	Parametric models (VAR and ARMAX models) with lower model complexities were shown to compete with a nonparametric method to automatically assess CA.
Jajcay and Hlinka. (2023)	Healthy participants	VAR	Resting-state EEG signals	Fitting VAR model to the EEG data demonstrated that microstate properties predominantly rely on the linear structure of the underlying EEG data
Kamiński et al. (1997)	Healthy participants	VAR	Resting-state EEG signals	The utilization of the VAR model for simultaneous assessment of EEG signals enabled exploration into brain synchronization and functional relationships
Lie and van Mierlo. (2017)	Surgical patients	VAR based on Kalman filter	Intracranial EEG signals	Kalman filter-based VAR models show the ability to conduct adaptive, multivariate functional-connectivity analyses on high-dimensional time-series EEG data
Nadalizadeh et al. (2023)	Healthy participants	VAR, VARMA, dual Kalman filter	Resting- and fatigue-state EEG signals	For classification between the resting- and fatigue state EEG signals, VAR model was fitted to selected sources to create state-space model, dual Kalman filter allowed estimation of dynamic source activity over time and their interrelations. Finally, VARMA model was fitted between EEG and source activity signals before feature selection and classification processes took place
Oikonomou et al. (2007)	Epilepsy patients	TVAR, Kalman filter	Resting-state EEG signals	Kalman filter was used to estimate the time-varying coefficient for the TVAR model. TVAR model was used for enhancement of the spikes in the EEG recordings achieving improvement in the signal-to-noise ratio and considerable decrease in the number of false positives
Omidvarnia et al. (2011)	Neonates	TVAR, dual extended Kalman filter	EEG signals	Dual extended Kalman filter was used for estimation of TVAR model parameters. TVAR models were compared in their ability to detect rapid changes in the cortical connectivity between EEG channels
Pagnotta and Plomp. (2018)	Rats	VAR, Kalman filter	Epicranial EEG signals	The study showed that when properly tuned, Kalman filter-based algorithms can model multivariate brain time series and reveal dynamic interaction patterns
Pascucci et al. (2020)	Rats	Self-tuning optimized Kalman filter	Epicranial EEG signals	The suggested model demonstrated its capability to monitor swiftly evolving patterns of directed connectivity within multivariate non-stationary time-series EEG signals
Pinto et al. (2022b)	Severe TBI patients	VARFI	ICP, CPP, ECG Other: ABP, MAP, EtCO_2_	The effectiveness of the VARFI model in estimating Transfer Entropy was shown, demonstrating its capability in evaluating the overall impact of long-term correlations and maintaining reliability when applied to short-time series
Rajabioun et al. (2017)	Healthy children	Dual Kalman filter	Resting-state EEG signals	The dual Kalman filter was employed to estimate effective connectivity among active regions concurrently
Samdin et al. (2013)	Healthy participants	TVAR	Task-state EEG signals	TVAR model was employed for dynamic classification of single-trial EEG signals, showing improvement in classification accuracy when compared to hidden Markov Model
Schäck et al. (2018)	TBI patients	Robust time-varying generalized partial directed coherence with Kalman filter, dual extended Kalman filter	ICP, PbtO_2_ Other: MAP	Both models were employed and contrasted to quantify the relationship between concurrently observed time series and unveil interactions among the signals with dual extended Kalman filter model showing slightly higher computational time
Swiercz et al. (1998)	Patients with intracerebral hemorrhage or brain tumor	Kalman filter	ICP	Kalman filter was compared with ANN and ARX for predicting ICP trends and detecting unfavorable symptom configurations, where ANN achieved superior prediction accuracy
Swiercz et al. (2000)	Patients with intracerebral hemorrhage or TBI	AR with Kalman filtering	ICP	AR with Kalman filtering was compared to ANN for prediction of on-line ICP where ANN achieved the best prediction accuracy
Thelin et al. (2020)	Mild to severe TBI patients	VARMA, VARIMA	ICP, PRx Other: ABP, MAP	Impulse response function plots were derived from the VARMA-derived coefficients. VARIMA model was used for assessment of the ICP and MAP relations over a minute interval
Zeiler et al. (2020)	Moderate to severe TBI patients	VARIMA	ICP, CPP Other: ABP, MAP	VARIMA and impulse response function plots used for assessing relationships between ICP and MAP during pre-, immediate post- and beyond post-decompressive craniectomy in TBI patients showing no variations between pre- and post-decompressive craniectomy
Zeiler et al. (2021)	Moderate to severe TBI patients	VARIMA	ICP, PbtO_2_ Other: ABP, MAP	VARIMA generated impulse response function plots were used to assess relationship between slow wave fluctuations in ICP, MAP and PbtO_2_ showing strong directional relation between MAP and ICP.

ABP, arterial blood pressure; ANN, artificial neural network; AR, autoregressive; ARMAX, autoregressive moving average with exogenous input; ARX, autoregressive with exogenous input; CBFv, cerebral blood flow velocity; CPP, cerebral perfusion pressure; ECG, electrocardiography; EEG, electroencephalography; EtCO_2_, end-tidal carbon dioxide; ICP, intracranial pressure; MAP, mean arterial pressure; PbtO_2_, brain tissue oxygenation; PRx, pressure reactivity index; rSO_2_, regional cerebral oxygen saturation; TBI, traumatic brain injury; TVAR, time-varying vector autoregressive; VAR, vector autoregressive; VARFI, vector autoregressive fractionally integrated; VARIMA, vector autoregressive integrated moving average; VARMA, vector autoregressive moving average.

## 4 Limitations of linear multivariate state-space models

While linear multivariate state-space models offer valuable insights and tools for analyzing complex systems, they also come with several inherent limitations effecting model selection, applicability, and interpretation various contexts. Many of these models assume linearity and stationarity of underlying processes, which may not hold true for many real-world systems exhibiting nonlinear and non-stationary behavior (Loaiza-Maya and Nibbering, 2023). Parameter estimation in multivariate state-space models can be challenging, particularly for high-dimensional data or complex systems, leading to potential biases in model predictions (Hinrichsen and Holmes, 2009). Sensitivity to initial conditions and limited flexibility in capturing complex interactions further constrain the utility of these models (Triantafyllopoulos and Triantafyllopoulos, 2021). Moreover, the computational complexity of analyzing and fitting multivariate state-space models, coupled with the risk of model overfitting and challenges in interpretability, poses significant hurdles in their application (Gessell et al., 2021; Triantafyllopoulos and Triantafyllopoulos, 2021). However specific models retain their own limitations and disadvantages.

The main limitation shared among the autoregressive models is the requirement for stationary data, which affects VAR, VARFI, VARFIMA, VARMA, and VARIMA models (Hinrichsen and Holmes, 2009; Triantafyllopoulos and Triantafyllopoulos, 2021). This assumption may not hold true in real-world datasets, potentially biasing parameter estimates and leading to unreliable forecasts. However, this limitation could be addressed through techniques such as differencing or, in some cases, incorporating fractional integration, i.e., VARFI and VARFIMA, with careful consideration during model specification and estimation. Additionally, all autoregressive models face challenges in dealing with high dimensionality, posing difficulties in accurate parameter estimation, and increasing the risk of overfitting (Triantafyllopoulos and Triantafyllopoulos, 2021), particularly in cerebral physiologic datasets, which often exhibit high dimensionality with numerous variables recorded simultaneously. Computational complexity is another common limitation, especially with increasing variables and lags, demanding substantial computational resources. Model interpretation complexity arises due to the intricate relationships among variables, requiring additional statistical techniques or domain knowledge (Olson et al., 2020).

Furthermore, all models have a limited forecasting horizon, typically suited for short-to medium-term predictions, and extrapolating beyond observed data may lead to unreliable forecasts, particularly if underlying relationships change over time (Olson et al., 2020; Triantafyllopoulos and Triantafyllopoulos, 2021). Additionally, careful model specification is crucial across all models to avoid bias in parameter estimates and inaccurate forecasts (Gessell et al., 2021). The limitations of the Kalman filter include its reliance on specific model assumptions such as linearity and Gaussian noise, which if violated, can lead to biased estimates (Maybeck et al., 1990). Moreover, its sensitivity to model mismatch, computational complexity, and the challenge of accurate initialization can hinder its performance, especially in complex systems (Kim et al., 2019; Sun et al., 2008). Tuning parameters and the assumption of complete observability further contribute to its limitations, along with its susceptibility to outliers and limited forecasting capability (Maybeck et al., 1990).

Additionally, cerebral physiologic data present unique challenges for multivariate state space modeling due to several factors. Apart from high dimensionality of the data, constructing appropriate multivariate state space models for cerebral physiologic data requires making assumptions about underlying physiological processes and interactions, which may not always hold true, leading to model misspecification and potential biases. Parameter estimation in such models is also challenging, especially with nonlinearities or non-Gaussian distributions in the cerebral physiologic data. Moreover, the complexity of multivariate state space models can hinder their interpretability, making it difficult to relate estimated parameters to underlying physiological mechanisms. Validation of these models is further complicated by the limited availability of ground truth measurements, risking overfitting and poor generalization performance. Finally, handling missing data and noise in cerebral physiologic datasets is crucial for accurate inference, as is addressing inter-subject variability stemming from factors like age, gender, and pathology.

## 5 Conclusion

This narrative review aimed to explore the significance of multivariate time-series analysis in understanding cerebral physiology. These analyses offer insights into the spatial and temporal dynamics of cerebral signals, aiding in the study of brain interactions, functional connectivity patterns, and detection of early signs of neurological disorders. Multivariate models, such as VAR models and state-space models, capture dynamic interactions, temporal relationships, and hidden states within cerebral signals, facilitating the development of trajectory models and clinical decision-making processes. Moreover, these models enable the integration of high-resolution cerebral data, reduce data overload, enhance signal quality, and provide valuable information about brain network dynamics and cerebrovascular dynamics. Additionally, they offer integrability into deep learning models, further enhancing their capabilities for analyzing cerebral physiology. By focusing on traditional statistical methodologies, such as multivariate AR models and the Kalman filter, this review aimed to bridge the gap between theoretical concepts and practical applications, offering a comprehensive understanding of their implications in cerebral physiology.
